# Association between mental health, psychological characteristics, and motivational functions of volunteerism among Polish and Ukrainian volunteers during the Russo-Ukrainian War

**DOI:** 10.1038/s41598-023-47840-z

**Published:** 2023-11-25

**Authors:** Agata Chudzicka-Czupała, Soon-Kiat Chiang, Clara M. Tan, Nadiya Hapon, Marta Żywiołek-Szeja, Liudmyla Karamushka, Mateusz Paliga, Zlatyslav Dubniak, Roger S. McIntyre, Roger Ho

**Affiliations:** 1 Faculty of Psychology, SWPS University, Chodakowska 19/31, 03-815 Warsaw, Poland; 2 Department of Psychological Medicine, Yong Loo Lin School of Medicine, National University of Singapore, Singapore, Singapore; 3 Department of Philosophy and Psychology, Ivan Franko National University of Lviv, Lviv, Ukraine; 4 G. S. Kostiuk Institute of Psychology, National Academy of Educational Sciences of Ukraine, Kyiv, Ukraine; 5 Faculty of Social Sciences, Institute of Psychology, University of Silesia, Katowice, Poland; 6 Department of Psychiatry, University of Toronto, Toronto, ON Canada; 7 Brain and Cognition Discovery Foundation, Toronto, ON Canada; 8 Institute for Health Innovation and Technology (iHealthtech), National University of Singapore, Singapore, Singapore

**Keywords:** Human behaviour, Quality of life

## Abstract

The Russo-Ukrainian War has led to a humanitarian crisis, and many people volunteered to help affected refugees. This cross-sectional survey study investigates the relationships between the psychological impact of participation, coping mechanisms, and motivational functions of volunteering during the Russo-Ukrainian War among 285 Ukrainian and 435 Polish volunteers (N = 720). Multivariate linear regression was used to examine relationships between motivational functions and psychosocial and demographic characteristics. Ukrainian volunteers reported significantly higher Hyperarousal and Avoidance, Depression, Anxiety, and Stress, Problem-focused, Emotion-focused, and Avoidant coping, as well as total scores of Hardiness and Psychological Capital than Polish counterparts. Linear regression analysis found that Impact of the Event Scale results, Coping with Stress, being a female, unemployed, and religious were significantly associated with higher motivational functions. Ukrainian volunteers could significantly reduce negative feelings and strengthen social networks and religious faith by volunteering, while Polish volunteers were significantly more likely to gain skills and psychosocial development from helping others.

## Introduction

Volunteerism is defined as a conscious, voluntary, and uncoerced activity for the benefit of others, going beyond family and friendship ties, for which the volunteer does not receive pay or any other form of material compensation, and which brings benefits both to the volunteer and to society at large^1^. Historically, volunteerism was observed in major wars. Many volunteers were involved in relief efforts during World War^2^. Even during the post-war period, volunteerism persisted in countries such as Britain. Numerous people continued to volunteer to learn, teach and administer first aid^3^. Volunteerism in wars has stood the test of time, with the recruitment of foreign war volunteers during ongoing wars such as the Syrian civil war that has been raging since 2011 and the conflict in eastern Ukraine.

### Volunteerism during the Russo-Ukrainian war

The ongoing Russo-Ukrainian war has led to humanitarian crisis among the Ukrainians. According to the United Nations High Committee on Refugees, the Russo-Ukraine war displaced more than 3 million people during the first year of the war. The number of refugees escalated in the second year^4^. Most refugees have gone to neighboring countries of Poland, Slovakia, Hungary, Romania, and Moldova, with most refugees fleeing to Poland. As of April 16, 2023, approximately 1.6 million were reported to have fled to Poland^5^, while 6 million Ukrainians were reported to be internally displaced. Before they are accepted as refugees in the neighboring countries, there is a period where they are in “a state of limbo.” During this period, they are left with uncertainty regarding their future, which greatly affects their psychological well-being^6^. While this may be temporary, the residual effect on their psyche may persist long after obtaining their refugee status.

In Poland, there are refugee centers at the border for Ukrainian refugees. The challenges faced by Ukrainian refugees include medical problems (e.g., infectious diseases, wounds), psychological trauma, and safety issues (e.g., human trafficking)^7^. The large influx of refugees into Poland may also greatly strain the government and relief agencies mobilized to help them. While the citizens of these neighboring countries accept them, integration into society and building a new life have not been easy. Many refugees in Ukraine are separated from their loved ones and may struggle to make ends meet in a new and foreign environment. They also battle the guilt and backlash from Ukrainians for abandoning their country in times of hardship^8^. Furthermore, many of these refugees arrive without medical records or an adequate supply of their medications^9^. A survey of older Ukrainian refugees in Moldova found that 28% required urgent medications for diabetes, hypertension, and pain relief, but over one-third could not afford them. Hence, this poses a challenge to managing those with pre-existing chronic conditions^10^ effectively.

Refugees in Ukraine faced similar challenges of lacking humanitarian and medical resources due to logistic issues. Long-standing issues such as underfunded healthcare and inequities in the distribution of healthcare resources had already been present before the Russo-Ukrainian war. While efforts have been in place to improve such problems, any gains related to healthcare improvements over the years are likely to be reversed due to the effects of the war^11^. A study to evaluate the impact of the Russia-Ukraine war on the citizens and the subsequent humanitarian crisis showed that, in addition to a large number of civilian casualties, at least 32 major hospitals in Ukraine were attacked during the study period. This crisis has significantly disrupted healthcare and health services, thus worsening citizens’ access to medical care and medications, especially those battling chronic diseases such as HIV and tuberculosis^11^.

Observing how the Russo-Ukrainian war has resulted in numerous civilian casualties and the destruction of major infrastructure sparked a sudden social spurt of international and local volunteering for refugees. For example, many Polish citizens have engaged in various voluntary services in Poland. Free accommodation was provided to the refugees crossing the border by owners of hotels and hostels along the borders. Tens and thousands of volunteers filled their private vehicles with essential items such as food, medications, thermal blankets, and diapers. They crossed the Ukrainian borders to pass supplies to the refugees waiting to enter Poland. Some have set up help points for refugees to receive essential daily items that Polish citizens have donated^12^. Many others have offered their time and service at refugee centers to help assist refugees in various ways, such as searching for jobs, financial assistance, or even providing psychological or legal support. A study done to analyze the experiences and motivation of the Polish volunteers who supported Ukrainian refugees found that most of the volunteering is primarily motivated by innate altruism and empathy to those in need rather than for personal enhancement (such as feeling better about oneself), religious or political reasons^13^.

Despite the risk that fighting in the war entails, more than 20,000 fighters from 52 countries have responded to the Ukrainian president’s plea for international volunteers and volunteered to serve in the Ukrainian military^14^. Even in Ukraine, volunteer movements started as early as the beginning of the Russo-Ukrainian war in 2014 with the annexation of Crimea. Over the years, thousands of Ukrainian citizens engaged in daily acts of care and service for people around them and the Ukrainian armed forces while living under wartime conditions themselves. By acting in daily solidarity and care, Ukrainians can act as agents of change and promote the importance of strengthening the society’s foundation to foster a stronger and more united country^15^. Moreover, organizations such as the Ukrainian-Israeli medical aid organizations have set up mobile clinics in villages and towns near the front lines after the war damaged some of the village’s health clinics^16^. Even three psychosocial centers in Ukraine have been opened up by the International Alert, in collaboration with the Global Initiative on Psychiatry, to assist those severely affected by the conflict in reintegrating into society. In addition, it has been active in peace-educating young people and children about trauma and violence^17^.

In this time of crisis, volunteerism is more important than ever. The motivation to volunteer is an interplay between altruism and self-interest in a situation (e.g., the victims' well-being during the Russo-Ukrainian war)^18^. The motivation to engage in volunteering is most often described according to the approach developed by Clary and Snyder^19^, referring to the function of the activity undertaken. It assumes that the phenomenon of volunteering can be analyzed in terms of discrepancies in the motives that are satisfied, the demands that are met, and the goals which the individual believes to be achieved^20^. The most frequently used questionnaire for measuring various motivations for volunteering along the altruism-egoism continuum is Volunteer Functions Inventory (VFI)^20^. Domaradzki et al.^13^ found that the majority of volunteers during the Russo-Ukrainian war emphasized that feelings of anger and compassion had influenced their decision to volunteer. The situation that occurred as a result of the outbreak of war in Ukraine was the reason to study the relationship between the psychological impact of taking part in volunteering for refugees, depression, anxiety, coping with stress mechanisms, and volunteer functioning motivational functions underlying volunteer activity during the Russo-Ukrainian War. It is important to add that research into the psychological effects of disasters has focused mainly on the victims, and only a few studies discuss the psychological consequences of volunteering for victims during such events^21^. Similarly, few empirical works have addressed the psychological consequences of volunteering in wartime circumstances resulting from providing aid to war victims^22^. The presented study makes it possible to fill this gap by considering variables such as anxiety, depression, post-traumatic stress symptoms, and coping with stress related to helping refugees. This inclusion is much needed, as the outbreak of war and the situation in Ukraine are traumatic and highly threatening. Media coverage concerning this, contact with individuals who have lost their loved ones and were forced to migrate and abandon their previous lives, can be a source of deterioration of the psychological condition of volunteers. In fact, research performed in natural disasters shows that volunteering in the humanitarian sector and helping disaster victims may be associated with a high likelihood of negative mental health consequences^23^. In addition to being exposed to traumatic situations, feeling threatened and afraid about their safety, and facing demanding working conditions, volunteers complain about negative reactions from the persons they help, lack of sleep, long working hours, being separated from their loved ones, interpersonal conflicts within the team, as well as material, financial difficulties^24,25^.

Omoto and Snyder^26^ point out that volunteering activity depends to a large extent on personality traits, which lead people to offer volunteering services. That is why variables that we controlled were the psychological capital^27^ and the mental hardiness of the volunteers^28^. Psychological capital is a hidden variable that includes self-efficacy, optimism, hope and resilience. Research confirms their importance for motivation and work engagement, proactivity, the use of effective problem-solving strategies, and mental health^27^. Hardiness has been described by the authors of the concept as a personality dimension, constituting a source of resilience and making it possible to treat life changes as challenges. Hardiness leads to involvement in what one does (commitment), the belief that the individual has an influence on the events forming their life (control), and the perception of change as a stimulus for one’s development (challenge)^28^. The relevance of these traits for volunteering activity has not been studied yet, so their inclusion in the model may lead to interesting findings. In addition to examining the severity of stress related to helping refugees the coping strategies undertaken were studied. These factors may translate into the individual feeling of fulfilment in volunteering activity.

Based on the conceptual framework of volunteering^29^, and because research has shown the significance of health and different personality traits in volunteer activity^30^, this study aimed to explore the possible associations between socio-demographic variables, mental health outcomes, coping with stress, psychological characteristics, such as hardiness (commitment, control, challenge), the psychological capital (hope, self-efficacy, optimism, resilience) and motivational functions of volunteerism for refugees among Poles and Ukrainians during the current Russo-Ukrainian war. Due to the multifaceted nature of the analyses and the fact that it is a new area of studies, the possible associations between the studied variables were researched in an exploratory manner.

Along with the exploration of the hypothesized relationships, the level of each variable was compared between Polish and Ukrainian volunteers. The relevance of these psychological traits and characteristics for volunteering activity during the war has yet to be studied, so their inclusion in the model may lead to interesting findings. Polish participants were chosen as a comparator group because they were bystanders in proximity to the conflicted area. Therefore, we formulated the following null hypothesis:

#### H0

*There are no statistically significant differences in the levels of post-traumatic stress, anxiety, depression, stress, coping with stress, psychological characteristics, such as hardiness (commitment, control, challenge), psychological capital (hope, self-efficacy, optimism, resilience) and motivational functions of volunteering between Ukrainian and Polish volunteers.*

This study will help to fill the existing gap in the literature by creating a more holistic model of volunteer engagement, which considers factors on which research has yet to be focused. We believe it is a step towards building a system for looking after volunteers' well-being and supporting them, which may translate directly into their commitment.

## Methods

### Design, study procedure, and participants

The study was conducted in two countries (Poland and Ukraine) from November 7 to December 30, 2022. Potential respondents were electronically invited in light of the ongoing conflict in Ukraine. The request for the distribution of the questionnaire and participation in the study was addressed to organizations engaged in volunteer work for Ukrainian refugees during the Russo-Ukrainian war. Information about this study and survey was posted on social media (e.g., Facebook, LinkedIn, Twitter, Telegram, and Viber). The survey was conducted via two online platforms (i.e., Google Forms Online Survey on social media and the SWPS University SONA platform). Online informed consent was obtained from all participants, and the data collected were anonymized and kept confidential. No incentives were offered to participants. The inclusion criteria for all participants were Polish or Ukrainian citizenship and aged between 18 and 70 years. To qualify to be volunteers, participants had to participate in volunteering activities to help Ukrainian refugees at least two times since the onset of the Russo-Ukrainian war. There were 1022 participants who completed the survey. Out of 1022 participants, the study used only data obtained from 720 volunteers who acted systematically and repeatedly participated in relief actions for refugees. The remaining 302 people, whose volunteer participation was episodic, were excluded from the sample.

### Measurements of demographics and motivational functions of volunteering

This study used the 2022 Russo-Ukrainian war refugee volunteering questionnaire developed by study team members in Ukraine and Poland. The questionnaire consisted of questions related to (1) Socio-demographic data and health status; (2) Information about their volunteering; (3) Help-seeking behavior during volunteer involvement; (4) The 30-item Volunteer Function Inventory (VFI) was used to measure the motivations for volunteering for Ukrainian refugees. The VFI assesses six volunteer functions, including (i) Value (the individual volunteers to express or act on important values like humanitarianism, “I can do something for a cause that is important to me”); (ii) Understanding (the volunteer is seeking to learn more about the world or exercise skills that are often unused, *“Volunteering lets me learn things through direct, hands on experience”*); (iii) Enhancement (one can grow and develop psychologically through volunteer activities, *“Volunteering makes me feel better about myself”*); (iv) Career (the volunteer has the goal of gaining career-related experience through volunteering, *“Volunteering allows me to explore different career options”*); (v) Social (volunteering allows an individual to strengthen his or her social relationships, *“Volunteering is an important activity to the people I know best*”) and vi) Protective (the individual uses volunteering to reduce negative feelings, such as guilt, or to address personal problems, *“No matter how bad I've been feeling, volunteering helps me to forget about it”*)^20^. The VFI tool was supplemented with an additional Religiosity scale to measure faith-related volunteer functions (5 items assessing to what extent volunteering is an activity driven by an individual's faith values and imperatives were added, *“I engage in volunteering for religious reasons”*). Respondents answered each item on a 7-point scale ranging from 1 (not at all important/accurate) to 7 (extremely important/accurate).

### Measurements of mental health outcomes, coping, and psychological characteristics

The 22-item Impact of Event Scale-Revised (IES-R) is a self-report questionnaire that corresponds to Diagnostic Statistical Manual Version IV (DSM-IV) symptoms of post-traumatic stress disorder (PTSD). For each item, respondents indicated the severity of self-perceived symptoms in the past week on a Likert scale from 0 (not at all) to 4 (extremely)^31^. The IES-R was not intended to diagnose PTSD but to assess subjective distress and PTSD symptoms^32^.

The 21-item Depression, Anxiety, and Stress Scales (DASS-21) were used to measure the volunteers' mood status^33^. The total DASS-21 score was used for analysis based on previous research^34^.

The 10-item Perceived Stress Scale (PSS-10) measured volunteers' stress levels^35^. In contrast, the 28-item Coping Orientation to Problems Experienced Inventory (Brief-COPE) was used to measure effective and ineffective ways to cope with the stressful life events of volunteers^36^. The three subscales include problem-focused coping, emotion-focusing coping, and avoidant coping.

The revised Norwegian Resilience (Hardiness) Scale (DSR-15R) was used to measure the general hardiness dimension and three sub-dimensions (commitment, control, and challenge) of the volunteers^37^. The Short Psychological Capital Questionnaire (PsyCap) assessed the volunteers' psychological capital, including hope, self-efficacy, optimism, and resilience^38^.

Previously prepared Ukrainian versions of selected tools were used^34^. Some tools were translated into Polish (VFI) and Ukrainian (VFI, PSS-10, DSR-15R, PsyCap) for the purpose of this study following the back-translation procedure used for psychosocial questionnaires proposed by Brislin^39,40^. The original items of the measures were independently translated by the authors of this study and then translated back to their English versions by Polish and Ukrainian experts in the English language. The next step was to discuss the differences that occurred, and an unanimous consensus was achieved. For all scales, Cronbach's alphas were satisfactory for group analyses (see Table 1).

**Table 1:** Cronbach’s alpha coefficients for Polish and Ukrainian language versions of the measures.

Measures	Cronbach’s alpha coefficients	
	Poland	Ukraine
VFI		
Understanding	0.86	0.87
Career	0.89	0.89
Values	0.77	0.75
Protective	0.86	0.83
Social	0.86	0.86
Enhancement	0.87	0.84
Religiosity	0.93	0.95
IES-R		
Intrusion	0.84	0.89
Hyperarousal	0.83	0.79
Avoidance	0.82	0.78
DASS-21		
Depression	0.87	0.88
Anxiety	0.89	0.88
Stress	0.89	0.87
PSS-10	0.80	0.79
Brief-COPE		
Problem-focused coping	0.85	0.75
Emotion-focused coping	0.71	0.75
Avoidant coping	0.81	0.71
DSR-15R		
Commitment	0.68	0.68
Control	0.80	0.77
Challenge	0.69	0.68
PsyCap		
Self-efficacy	0.86	0.84
Hope	0.78	0.76
Resilience	0.70	0.68
Optimism	0.83	0.86

*VFI* Volunteer Function Inventory, *PSS-10* Perceived Stress Scale, *IES-R* Impact of Event Scale-Revised, *DASS-21* Depression, Anxiety, Stress Scale, Brief-COPE Brief Coping Orientation to Problems Experienced questionnaire, *DSR-15R* Norwegian Dispositional Resilience (Hardiness) Scale, *PsyCap* Positive Psychological Capital questionnaire.

### Statistical analysis

Categorical variables were presented as percentages of responses to the questions, calculated based on the number of respondents per response to the total number of responses. The Chi-square test was used for the comparison of the categorical variables. The total scores for VFI, IES-R, DASS-21, PSS-10, Brief-COPE, DSR-15R, PsyCap, and their respective subscales were expressed as mean and standard deviation. The Student’s t-test was used to compare the differences in the psychosocial mean scores between the Ukrainian and Polish participants. Multivariate linear regression was used to calculate the association between VFI scores with the psychosocial scores and demographic characteristics for both populations and the whole sample. All tests were two-tailed, and a significance level of *p* < 0.05 was used. The statistical analysis was done in SPSS Statistic 28.0.

### Ethics approval

The study was conducted in accordance with the Declaration of Helsinki and approved by the Institutional Review Board of SWPS University, Poland (WKEB81/01/2023).

### Informed consent statement

Informed consent was obtained from all subjects involved in the study. Subjects are all above the age of 18 years, so no informed consent was needed from parents or guardians.

## Results

### Comparison of demographic data and psychosocial profile

This study had 720 volunteers (Poland: 435; Ukraine: 285). The sociodemographic data of the volunteers are summarized in Table 2. The mean age of Polish and Ukrainian volunteers was 26.54 ± 8.85 and 30.13 ± 10.60 years, respectively. Majority of volunteers were women; had a university education; are religious; without chronic illness; presented at least average self-health rating; suffered from COVID-19 in the past 3 years; without past psychiatric history and without exposure to serious accident, life-threatening condition or disaster before the war; would likely volunteer for Ukrainian refugees again in the future; spend less than 2 h on media news-related to helping Ukrainian refugees; and had at least average sleep quality since volunteering. Little over half of Polish volunteers agreed that media exposure to the current war in Ukraine is psychologically traumatic, while just over one-third of Ukrainian volunteers agreed with it.

**Table 2:** Socio-demographic characteristics of the Polish and Ukrainian volunteers (N = 720).

Socio-demographic characteristics	Mean ± SD (Number (%))		
	Poland (N = 435)	Ukraine (N = 285)	*p*-value
Gender			
Female	379 (87.1)	221 (77.5)	0.005**
Male	51 (11.7)	64 (22.5)	
Different	5 (1.2)	0 (0)	
Age	26.54 ± 8.85	30.13 ± 10.60	< 0.001***
Education attainment			
Primary school	0 (0)	6 (2.1)	< 0.001***
Secondary school	259 (59.5)	57 (20)	
Bachelor’s	64 (14.7)	106 (37.2)	
Postgraduate	112 (25.7)	116 (40.7)	
Marital status			
Single	143 (32.9)	87 (30.5)	0.046*
Married	79 (18.2)	116 (40.7)	
In a relationship with a significant other	199 (45.7)	63 (22.1)	
Divorced/Separated	14 (3.2)	18 (6.3)	
Widowed	0 (0)	1 (0.4)	
Employment status			
Student	242 (55.6)	92 (32.3)	< 0.001***
Employed	180 (41.4)	171 (60)	
Unemployed	4 (0.9)	9 (3.2)	
Homemaker	7 (1.6)	7 (2.5)	
Farmer	0 (0)	3 (1.1)	
Retired	2 (0.5)	3 (1.1)	
Parental status			
No children	349 (80.2)	176 (61.8)	< 0.001***
I have a child/children 15 years old or younger	62 (14.3)	56 (19.6)	
I have a child/children older than 15 years	24 (5.5)	53 (18.6)	
Household size			
One person	72 (16.6)	18 (6.3)	< 0.001***
Two people	129 (29.7)	41 (14.4)	
Three to five people	222 (51)	193 (67.7)	
Six people or more	12 (2.8)	33 (11.6)	
Religious (Yes)	218 (50.1)	242 (84.9)	< 0.001***
Refugee (Yes)	0 (0)	21 (7.4)	< 0.001***
Chronic illness (Yes)	86 (19.8)	52 (18.2)	0.612
Self-rated health status			
Good	254 (58.4)	146 (51.2)	0.436
Fair	78 (17.9)	78 (27.4)	
Poor	103 (23.7)	61 (21.4)	
Had COVID-19 the past 3 years (Yes)	306 (70.3)	209 (73.3)	0.386
Past psychiatric history (Yes)	201 (46.2)	58 (20.4)	< 0.001***
Experienced a serious accident, life-threatening situation, or disaster (Yes)	124 (28.5)	90 (31.6)	0.378
Years of volunteer work	4.49 ± 5.30	3.81 ± 5.89	0.108
Involvement in helping war refugees from Ukraine			
Involved several times	337 (77.5)	123 (43.2)	< 0.001***
Involved more than a dozen times	60 (13.8)	96 (33.7)	
Involved continuously since the war outbreak	38 (8.7)	66 (23.2)	
My participation in volunteering for refugees from Ukraine			
Unlikely to happen again in the future	26 (6)	11 (3.9)	0.044*
Neutral	36 (8.3)	14 (4.9)	
Likely to happen again in the future	373 (85.7)	260 (91.2)	
Average time spent per day following media news-related to helping refugees from Ukraine			
0 h	110 (25.3)	48 (16.8)	< 0.001***
Up to 1 h	280 (64.4)	99 (34.7)	
1–2 h	29 (6.7)	92 (32.3)	
3–5 h	10 (2.3)	32 (11.2)	
6 h or more	6 (1.4)	14 (4.9)	
Media exposure to the current war in Ukraine is psychologically traumatic			
Disagree	70 (16.1)	87 (30.5)	< 0.001***
Neutral or no comment	114 (26.2)	93 (32.6)	
Agree	251 (57.7)	105 (36.8)	
Quality of sleep since volunteering for Ukrainian refugees			
Poor	20 (4.6)	23 (8.1)	0.018*
Average	269 (61.8)	185 (64.9)	
Good	146 (33.6)	77 (27)	
Mental healthcare professionals whom I seek help from			
Psychiatrist	169 (38.9)	55 (19.3)	< 0.001***
Clinical psychologist or counselor	225 (51.7)	155 (54.4)	
Other healthcare professionals (General practitioners, Social workers, Nurses etc.)	7 (1.6)	42 (14.7)	
Online psychotherapist	34 (7.8)	33 (11.6)	

*SD* standard deviation, *p < 0.05; **p < 0.01; ***p < 0.001.

### Comparison of levels of motivational functions of volunteering, depression, anxiety, stress, post-traumatic stress, coping, resilience, and other psychological characteristics

Table 3 shows the comparison of the VFI, IES-R, DASS-21, PSS-10, Brief COPE, DSR-15R, and PsyCap scores among Polish and Ukrainian volunteers. Ukrainian volunteers reported significantly higher VFI total scores (*p* < 0.01), protective, social, and religiosity scores (*p* < 0.001), whereas Polish volunteers reported significantly higher understanding and enhancement scores (*p* < 0.05). Sixty percent of Ukrainian and 53.8% of Polish volunteers had VFI scores above the mid-point. Ukrainian volunteers also had significantly higher IES-R total scores (*p* < 0.05), especially in hyperarousal (*p* < 0.001) and avoidance (*p* < 0.01) scores compared to Polish volunteers. In addition, Ukrainian volunteers had significantly higher DASS-21 total scores, depression, anxiety, and stress scores (*p* < 0.001). Furthermore, Ukrainian volunteers reported significantly higher scores in problem-focused, emotion-focused, and avoidant coping (*p* < 0.001). Lastly, significantly higher DSR-15R (*p* < 0.01) and PsyCap (*p* < 0.001) total scores were reported by Ukrainian volunteers.

**Table 3:** Psychosocial profile of the Polish and Ukrainian volunteers (N = 720).

Psychosocial profile	Mean ± SD (number (%))		
	Poland (N = 435)	Ukraine (N = 285)	*p*-value
**VFI score**	**125.82 ± 34.38**	**133.43 ± 40.72**	0.007**
Understanding	24.28 ± 7.02	22.69 ± 8.05	0.005**
Career	14.74 ± 8.04	15.03 ± 8.41	0.647
Values	27.50 ± 5.01	28.26 ± 5.52	0.055
Protective	14.53 ± 7.54	16.68 ± 7.98	< 0.001***
Social	15.30 ± 7.05	17.61 ± 7.69	< 0.001***
Enhancement	20.79 ± 7.60	19.31 ± 8.07	0.013*
Religiosity	8.69 ± 6.33	13.85 ± 9.49	< 0.001***
**IES-R score**	**29.30 ± 15.93**	**32.39 ± 16.38**	0.012*
Intrusion	11.94 ± 6.31	11.73 ± 7.18	0.667
Hyperarousal	9.10 ± 5.70	11.09 ± 5.53	< 0.001***
Avoidance	8.25 ± 5.61	9.58 ± 5.40	0.002**
**DASS-21 score**	**34.09 ± 26.59**	**45.44 ± 26.29**	< 0.001***
Depression	10.13 ± 8.91	13.02 ± 9.57	< 0.001***
Anxiety	9.29 ± 9.48	13.45 ± 9.55	< 0.001***
Stress	14.66 ± 10.39	18.96 ± 9.92	< 0.001***
**PSS-10 score**	**15.97 ± 5.85**	**16.02 ± 5.31**	0.897
**Brief-COPE score**	**35.97 ± 12.27**	**41.99 ± 11.39**	0.022*
Problem-focused coping	13.89 ± 5.35	14.76 ± 4.38	< 0.001***
Emotion-focused coping	15.81 ± 5.63	19.65 ± 5.94	< 0.001***
Avoidant coping	6.26 ± 4.46	7.57 ± 4.12	< 0.001***
**DSR-15R score**	**28.53 ± 6.03**	**30.00 ± 6.34**	0.002**
Commitment	9.85 ± 2.77	10.69 ± 2.94	< 0.001***
Control	10.73 ± 2.85	11.20 ± 3.10	0.036*
Challenge	7.95 ± 2.72	8.12 ± 2.20	0.388
**PsyCap score**	**53.89 ± 9.30**	**56.91 ± 9.36**	< 0.001***
Self-efficacy	13.16 ± 3.09	13.56 ± 3.00	0.087
Hope	13.70 ± 2.74	13.81 ± 2.80	0.596
Resilience	14.14 ± 2.59	15.09 ± 2.58	< 0.001***
Optimism	12.89 ± 3.33	14.44 ± 3.03	< 0.001***

Significant values are marked with asterisks.

The global results of each tool are in bold.

*VFI* Volunteer Function Inventory, *PSS-10* Perceived Stress Scale, *IES-R* Impact of Event Scale-Revised, *DASS-21* Depression, Anxiety, Stress Scale, *Brief-COPE* Brief Coping Orientation to Problems Experienced questionnaire, *DSR-15R* Norwegian Dispositional Resilience (Hardiness) Scale, *PsyCap* Positive Psychological Capital questionnaire, *p < 0.05; **p < 0.01; ***p < 0.001.

In comparison to non-volunteers from both countries (N = 302), volunteers had significantly higher IES-R total scores (30.52 ± 16.17 vs. 28.13 ± 17.08; *p* < 0.05). Volunteers also reported significantly higher DASS-21 total scores (38.58 ± 27.03 vs. 33.67 ± 26.10; *p* < 0.01). In addition, volunteers had significantly higher problem-focused (14.24 ± 5 vs. 12.8 ± 5.79) and emotion-focused (17.33 ± 6.05 vs. 15.42 ± 6) scores (*p* < 0.001).

### Linear regression analysis with total VFI scores (motivation for volunteering) as the dependent variable

Table 4 shows the linear regression using VFI scores as the dependent variable and the other psychosocial scores as the independent variable. The linear regression analysis found that IES-R and Brief-COPE total scores were significantly associated with higher VFI scores for Poles and Ukrainian volunteers after adjusting other variables (*p* < 0.001).

**Table 4:** Linear regression analysis of VFI score against other psychosocial scores of the volunteers (N = 720).

Psychosocial profile	Poland (N = 435)		Ukraine (N = 285)		Combined (N = 720)	
	B (SE)	*p*-value	B (SE)	*p*-value	B (SE)	*p*-value
IES-R score	0.714 (0.144)	< 0.001***	0.719 (0.197)	< 0.001***	0.717 (0.116)	< 0.001***
DASS-21 score	− 0.032 (0.084)	0.700	− 0.182 (0.130)	0.162	− 0.090 (0.070)	0.198
PSS-10 score	− 0.536 (0.292)	0.067	− 0.245 (0.477)	0.608	− 0.421 (9.243)	0.097
Brief-COPE score	0.824 (0.142)	< 0.001***	1.194 (0.230)	< 0.001***	0.959 (0.122)	< 0.001***
DSR-15R score	− 0.028 (0.320)	0.931	0.244 (0.411)	0.554	0.113 (0.251)	0.652
PsyCap score	0.350 (0.209)	0.094	0.116 (0.292)	0.691	0.250 (0.168)	0.137

*VFI* Volunteer Function Inventory, *PSS-10* Perceived Stress Scale, *IES-R* Impact of Event Scale-Revised, *DASS-21* Depression, Anxiety, Stress Scale, *Brief-COPE* Brief Coping Orientation to Problems Experienced questionnaire, *DSR-15R* Norwegian Dispositional Resilience (Hardiness) Scale, *PsyCap* Positive Psychological Capital questionnaire, *p < 0.05; **p < 0.01; ***p < 0.001.

Table 5 shows the linear regression analysis results using VFI scores as the dependent variable and demographic characteristics as the independent variable. The linear regression analysis found that female gender, unemployment, and religion were significantly associated with higher VFI scores after adjusting other variables (*p* < 0.01). Interestingly, Polish volunteers with poor self-rated health status were significantly associated with higher VFI scores (*p* < 0.05) but not for Ukrainian counterparts.

**Table 5:** Linear regression analysis of VFI score against demographic characteristics of the volunteers (N = 720).

Demographic Characteristics	Poland (N = 435)		Ukraine (N = 285)		Combined (N = 720)	
	B (SE)	*p*-value	B (SE)	*p*-value	B (SE)	*p*-value
Gender (Female)	8.634 (4.714)	0.068*	14.758 (5.814)	0.012*	9.944 (3.566)	0.005**
Age	− 0.382 (0.378)	0.313	0.161 (0.428)	0.707	− 0.129 (0.272)	0.634
Education attainment	− 0.634 (3.294)	0.847	− 6.275 (3.412)	0.067	− 2.626 (2.145)	0.221
Marital status	− 3.965 (3.522)	0.261	− 2.566 (5.437)	0.637	− 3.946 (2.934)	0.179
Employment status^a^	− 10.178 (4.528)	0.025*	− 14.041 (5.959)	0.019*	− 10.506 (3.565)	0.003**
Parental status	3.449 (4.720)	0.465	2.218 (5.389)	0.681	3.980 (3.452)	0.249
Household size	2.903 (2.253)	0.198	− 1.076 (3.620)	0.767	2.671 (1.835)	0.146
Religious status	8.059 (3.269)	< 0.001***	21.353 (6.870)	< 0.001***	13.152 (2.941)	< 0.001***
Poor self-rated health status	3.830 (1.955)	0.028*	3.758 (3.012)	0.213	4.236 (1.644)	0.01*
Refugee status	NA		− 9.439 (9.282)	0.310	− 5.950 (8.089)	0.462
Chronic illness	0.123 (4.210)	0.977	0.815 (6.328)	0.898	− 0.387 (3.493)	0.912
Had COVID-19 the past 3 years	1.869 (3.580)	0.602	− 0.283 (5.437)	0.959	1.445 (2.997)	0.630
Past psychiatric history	− 1.990 (3.328)	0.550	0.034 (6.248)	0.996	− 2.392 (2.980)	0.422
Experienced a serious accident, life-threatening situation, or disaster	− 6.251 (3.584)	0.082	0.356 (5.192)	0.945	− 3.520 (2.959)	0.235

*p* < 0.05; **p < 0.01; ***p < 0.001.

^a^Recoded into Unemployed and Employed.

## Discussion

This is the first study comparing the extent of the motivational functions of volunteering, coping with stress, anxiety, depression, stress, and post-traumatic stress levels between volunteers from Poland and Ukraine during the Russo-Ukrainian war. The key findings are summarized as follows: Firstly, as expected, Ukrainian volunteers reported significantly higher scores for post-traumatic stress, anxiety, depression, and stress than Polish volunteers. Despite the adverse effects of the war on mental health, Ukrainian volunteers reported significantly higher scores in motivational functions of volunteering, including protective, social, enhancement, and religiosity. Coping with stress styles are the strategies and behaviors that volunteers might use to manage the demands and challenges associated with the Russo-Ukrainian war. Ukrainian volunteers reported significantly higher scores in problem-focused, emotion-focused, and avoidant coping; domains of resilience including commitment and control as well as positive psychological capital including resilience and optimism. Hence, the formulated null hypothesis can be rejected. Fifth, higher IES-R scores, higher Brief-COPE scores, female gender, and religiosity were significantly associated with higher motivational functions of volunteering.

The theory and conceptual models behind volunteerism are complicated, and no single and integrated theory can explain the factors associated with volunteerism^29^. During peaceful times, volunteerism can be seen as an investment in one’s human capital^41^. A sudden event, such as the Russo-Ukrainian war, may have a huge impact on levels of volunteering^42^. During the war, volunteer aid offered to at-risk populations can reduce the prevalence of psychiatric symptoms and suffering during times of conflict and violence^34,43^. Domaradzki et al.^13^ reported that the motivation to be volunteers was due to a general willingness to help and volunteer on behalf of Ukrainian refugees. As the Russo-Ukrainian war is unique and volunteering targets Ukrainians during the current crisis, the factors associated with motivational volunteer functions are expected to differ from other civilian situations (e.g., volunteer work in schools or elderly homes)^44^. The current war may attribute different meanings and functions to volunteerism. During wartime, volunteering can be viewed as social resources or social expression of support and sympathy for victims of the war. This study provides novel information about the mental well-being and individual-level factors associated with motivational volunteering functions in Ukraine and its neighbor, Poland. The results of our survey can be translated into practice. They allow the identification of difficulties experienced by volunteers and indicate the relationship of these factors to motivation, which is important for building psychological support systems for volunteers engaging in relief activities in similar difficult situations in the future. This finding can contribute to the development of civil society.

The VFI identifies seven personal and social functions served by volunteering: career (goal of obtaining career-related experience); enhancement (psychological development); protective (reduce negative feelings or personal problems); value (acting upon important personal values related to altruistic and humanitarian concerns for others); understanding (learn or exercise knowledge, skills use skills that may otherwise remain unused); social (strengthen or create social relationships and deal with concerns over social rewards and punishments); and religious (strengthen religious faith)^20^. There were significant differences in the motivational functions of volunteering between Polish and Ukrainian volunteers. Ukrainian volunteers reported significantly higher scores in protective, social, and religious volunteer functions. This finding suggests that Ukrainian volunteers could significantly reduce negative feelings and strengthen social networks and religious faith by volunteering during the Russo-Ukrainian war. In contrast, Polish volunteers reported significantly higher scores in motivation based on understanding and enhancement. This finding suggests that Polish volunteers were significantly more likely to engage in volunteerism to develop their skills and psychosocial resources during the Russo-Ukrainian war. There were no differences between Polish and Ukrainian volunteers in career and values goals. This finding suggests that volunteers from both countries shared common values and that the motivation to work for war refugees was unrelated to their careers.

Our findings support the conceptualization of volunteerism during the Russo-Ukrainian War as multi-dimensional^45^. This study identifies three dimensions that provide us with the essential building blocks for our conceptualization. First, the post-traumatic symptoms, as indicated by IES-R scores but not levels of anxiety or depression, correlate with the motivational functions of volunteering. Jobst et al.^46^ reported that around 57% of German refugee helpers had experienced a traumatic event, and around 3% of the helpers had a positive PTSD screening. Second, overall coping score but not resilience or psychological capital correlated with volunteering functioning. Third, female gender and religion are the only sociodemographic factors that correlate with the motivational functions of volunteering. The above findings were found in Polish and Ukrainian participants, which was unsurprising. Volunteering is collectively oriented in nature and represents a distinct type of social bond and social ties between Poles and Ukrainians^29^. The act of volunteering stood out as a primary expression of core human values such as altruism, compassion, democracy, justice, and social responsibility^47^. Coping with stress scores was positively associated with the motivational functions of volunteering. Volunteering provides distinctive opportunities like helping and prosocial actions^48^. Our findings correspond with observations made by a recent study on Polish volunteers during the Russo-Ukrainian War, which described helping Ukrainian refugees both as a moral imperative and a satisfying experience by increasing self-esteem and accomplishment^13^.

### Limitations and future studies

Although this is one of only a few studies conducted on the motivational functioning of Polish and Ukrainian volunteers during the Russo-Ukrainian War, this study has several limitations. Firstly, this study did not explore other factors related to the intensity of the motivational functions of volunteering, including economic and political factors, temperamental and other personality traits than resilience (hardiness) and traits that are part of psychological capital^29^. Motives are likely to be connected with other traits, such as internal or external locus of control, self-esteem, system of values or other individual features. It would be worth to study the role of social support volunteers get from others. Due to the above limitations, we do not consider our findings the ultimate theory behind volunteering in the Russo-Ukrainian War. Secondly, our study did not explore the negative aspects of volunteering, including burnout^49^. Thirdly, there were significantly more women than men in the sample. While this is consistent with statistics^50^, as more women engage in volunteering for refugees, it may be important due to gender differences in coping with stress and motivation.

Additionally, this study used anonymous questionnaires and was subjected to selection bias^46^. As a result, our sample might only partially represent part of the community of Polish and Ukrainian volunteers. Finally, we used questionnaires to assess symptoms of depression, anxiety, stress, and PTSD. The severity of symptoms were not equivalent to clinical diagnoses established by psychiatrists. This study measured immediate psychological impact, and further longitudinal study is required to monitor long-term psychological reaction on participation in volunteer activity. Future research should consider these limitations. It would be advisable to enrich the research and use a different methodology, including clinical interviewing, as well as qualitative methods that would give insight into the subjective experiences of people involved in relief efforts for war refugees.

Studies of volunteerism have just begun as the war is ongoing at the time of preparing this manuscript. Performing further research and understanding volunteerism is beneficial for the volunteers as well as the recipients. In conclusion, post-traumatic symptoms, total coping score, female gender, and presence of religious faith correlate with volunteering function during the Russo-Ukrainian war. Ukrainian volunteers could significantly reduce negative feelings, and strengthen their social network and religious faith by volunteering, while Polish volunteers were significantly more likely to aim for skills and psychosocial development by volunteering.

## Data Availability

The data presented in this study are available on request from the corresponding author.
